# The Changing Role of Gene-Expression Profiling in the Era of De-escalating Adjuvant Chemotherapy in Early-Stage Breast Cancer

**DOI:** 10.1245/s10434-019-07511-8

**Published:** 2019-06-17

**Authors:** J. E. C. van Steenhoven, A. Kuijer, K. Schreuder, S. G. Elias, P. J. van Diest, E. van der Wall, S. Siesling, T. van Dalen

**Affiliations:** 1 Department of Surgery, Diakonessenhuis Utrecht, Utrecht, The Netherlands; 2 Department of Pathology, University Medical Center Utrecht, Utrecht University, Utrecht, The Netherlands; 3 Department of Surgery, St. Antonius Hospital, Nieuwegein, The Netherlands; 4 Department of Research, Netherlands Comprehensive Cancer Organisation, Utrecht, The Netherlands; 5 Department of Epidemiology, Julius Center for Health Sciences and Primary Care, University Medical Center Utrecht, Utrecht University, Utrecht, The Netherlands; 6 Department of Medical Oncology, University Medical Center Utrecht, Utrecht University, Utrecht, The Netherlands; 7 Department of Health Technology and Services Research, Technical Medical Centre, University of Twente, Enschede, The Netherlands

## Abstract

**Purpose:**

We assessed the recent trends in the administration of adjuvant chemotherapy thereby evaluating the role of the 70-gene signature (70-GS) testing in decision-making in the systemic treatment of patients with lymph node negative (N0) and lymph node positive (N+) breast cancer.

**Methods:**

Patients with a national guideline directed indication for 70-GS use treated between 2013 and 2016 were selected from the Netherlands Cancer Registry. Time trends in the administration of adjuvant chemotherapy were evaluated within guideline- and age-delineated subgroups. The influence of the 70-GS on chemotherapy use was assessed with logistic regression.

**Results:**

During the study period, the overall administration of adjuvant chemotherapy decreased from 49 to 23% and 70-GS use increased from 24 to 51%. The 70-GS was not associated with a decreased likelihood for N0 patients to receive chemotherapy (odds ratio [OR] 1.0; 95% confidence interval [CI] 0.86–1.17), as the proportion of N0 patients who received chemotherapy in the absence of 70-GS use decreased during the study period. In patients with N1a disease, 70-GS testing was associated with a decreased likelihood to receive chemotherapy (OR 0.21; 95% CI 0.15–0.29). In patients < 50 years and 50–59 years of age, 70-GS use was associated with a consistent lower proportion of patients receiving chemotherapy throughout the study period (OR 0.17; 95% CI 0.13–0.23 and OR 0.53; 95% CI 0.43–0.65, respectively).

**Conclusions:**

In this population-based study, the administration of adjuvant chemotherapy in ER+ breast cancer strongly declined. For node-positive and younger patients, 70-GS use was associated with a decreased probability for patients to receive adjuvant chemotherapy.

**Electronic supplementary material:**

The online version of this article (10.1245/s10434-019-07511-8) contains supplementary material, which is available to authorized users.

Over the past two decades, the importance of tumor biology in relation to breast cancer outcome and the varying beneficial effect of adjuvant chemotherapy for the molecular cancer subtypes are increasingly recognized.^1^^,^^2^ Particularly in patients with estrogen receptor (ER)-positive (+) breast cancer, the routine use of adjuvant chemotherapy has been questioned in recent years.^3^^,^^4^ Gene-expression profiles (GEPs) were developed and validated for outcome prediction in ER+ early-stage breast cancer patients, and its use has been incorporated in both national and international breast cancer guidelines.^5^–^12^

The Dutch guideline of 2012 recommended to administer adjuvant chemotherapy in all lymph node-positive patients and in lymph node-negative patients with unfavorable clinicopathological characteristics (i.e., T2 grade I or T1c grade II tumors, and all grade III tumors).^13^ However, the same national guideline also suggested to consider the use of a validated GEP in patients with ER+/Her2− tumors of low or intermediate malignancy grade with no or limited metastatic lymph node involvement. In 2015, the St. Gallen expert panel was the first to reconsider the routine administration of chemotherapy in “Luminal A-like” breast cancer (i.e., HR+, Her2−, Ki-67 low or gene signature low risk), thereby questioning mere tumor size and involvement of one to three lymph nodes as criteria to warrant chemotherapy administration.^3^

In a previous nationwide study, we demonstrated that the use of the 70-GS was associated with a significant reduction of chemotherapy administration in the subset of ER+/Her2− disease without overt lymph-node metastasis (≤ Nmi) treated between 2011 and 2013.^14^ Recent randomized trials studying the contribution of GEPs to the decision to administer adjuvant chemotherapy also suggest a role for GEPs in lymph node-positive patients.^8^^,^^15^

In the present study, we describe the time trends in chemotherapy use in a large population-based cohort of ER+/Her2− breast cancer patients considered eligible for GEP use according to national guidelines, encompassing the period of time since the Dutch breast cancer guideline first suggested a role for GEP use until the period that the results of the GEP trials were available. Furthermore, the use and impact of the 70-GS on chemotherapy administration was evaluated in different subgroups delineated by lymph node status, grade, tumor size, and age.

## Patients and Methods

Data on patient, tumor, and treatment characteristics were derived from the Netherlands Cancer Registry (NCR). All Dutch female patients (> 17 years) surgically treated for primary unilateral invasive ductal breast cancer between January 2013 and December 2016 were identified in the NCR database. Patients with a prior history of malignancy or those who received neoadjuvant chemotherapy, were excluded from the analysis.

During the study period, the national guideline of 2012 was effective. According to this guideline, adjuvant chemotherapy should be administered to all patients with lymph node positive disease (≥ N1a) and to patients without lymph node involvement but with unfavorable clinicopathological features (all grade III tumors, grade II tumors > 1 cm, any tumor > 2 cm, or Her2+ tumors), as well as in patients of young age (< 35 years). This guideline also suggested the use of validated GEP in ER+ breast cancer, when there is doubt about the indication for adjuvant chemotherapy based on traditional clinicopathological risk factors.^13^ Patients with grade III tumors were not included, because these patients were not considered candidates for GEP use. Although the 70-GS and OncotypeDx are both commercially available in the Netherlands, OncotypeDx was rarely used during the study period.^16^ We therefore focused on the use and impact of 70-GS only.

We delineated four groups of patients < 70 years of age, suffering from ER+/Her2− invasive ductal breast cancer, who were considered eligible for GEP use based on the aforementioned guideline criteria. In addition, we included patients with macro-metastatic lymph involvement based on the more recently suggested role of GEP use in this subset of patients. The following four groups were composed: group A (pN0; grade I; > 2 cm), group B (pN0; grade II; > 1 cm), group C (pNmi, grade I/II, any size), and group D (pN1a, grade I/II, any size).

### Statistical Analysis

Frequencies of patient and tumor characteristics of patients eligible for GEP use (i.e., clinical intermediate risk) were compared between patients who received the 70-GS versus patients who did not receive the test, using a *χ*^2^ test for differences in categorical data. For normally distributed continuous variables (age and size), means were calculated and a *t* test was performed. For the whole group, the proportions of patients who received adjuvant chemotherapy, irrespective of GEP use, were calculated for the years 2013–2016. For the defined subgroups A–D, the proportions of patients in whom the 70-GS was applied were calculated and observed over time. Adherence to the test result in terms of the administration of chemotherapy in the overall study population and the aforementioned subgroups (A–D) was calculated by dividing the sum of patients with a low-risk test result in whom adjuvant chemotherapy was omitted and patients with a high-risk test result who received adjuvant chemotherapy by all patients with a known test result. The differences in chemotherapy administration between patients who received the 70-GS versus patients who did not receive the test were evaluated using a *χ*^2^ test or Fisher’s exact test when the proportions of patients within this category were small.

In addition, we investigated the association between 70-GS use on the administration of adjuvant chemotherapy within three different age categories (< 50 years, 50–59 years, and 60–69 years) using a *χ*^2^ test. Subsequently, logistic regression analysis was performed within the different guideline and age delineated subgroups to assess whether GEP use was independently associated with the administration of adjuvant chemotherapy after correction for clinicopathological confounders (age, grade, tumor size, N-status, PR status) and incidence year. Results are presented as odds ratios (OR) and accompanying 95% confidence intervals (95% CI). All tests were two-sided, and *P* value < 0.05 was considered to be statistically significant. All statistical analyses were performed in R (Version 3.2.1).

## Results

### Study Population

**Fig. 1:**
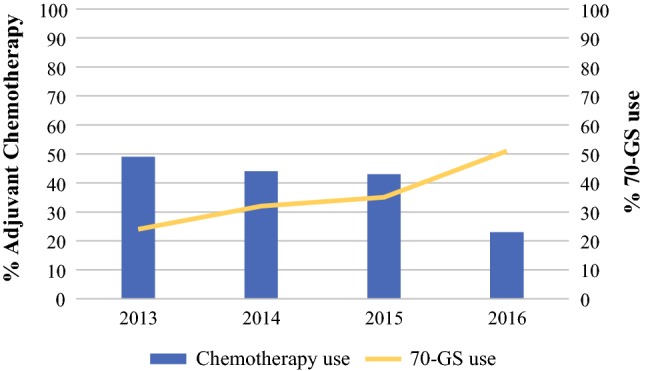
Time trend in the administration of adjuvant chemotherapy in the total study population eligible for gene-expression profiling and the use of the 70-gene signature

A total of 6780 breast cancer patients treated between 2013 and 2016 who were eligible for GEP could be identified in the NCR, of whom 281 patients (4%) were assigned to group A (BR I, > 2 cm, N0), 3571 patients (53%) to group B (BR II, > 1 cm, N0), 1040 patients (15%) to group C (BR I/II, any size, Nmi), and 1888 of patients (28%) to group D (BR I/II, any size, N1a). Chemotherapy was administered in 40% of all patients and decreased during the study period: in 2013, 49% of patients within the delineated indication area for GEP use received adjuvant chemotherapy versus 23% of patients in 2016 (Fig. 1).

**Fig. 2:**
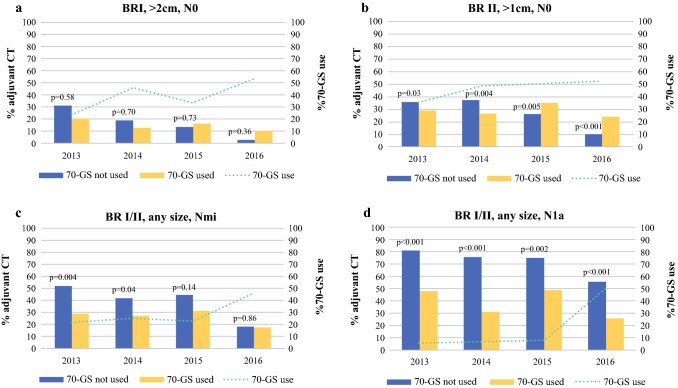
**a**–**d** Adjuvant chemotherapy use in patients who received the 70-gene signature versus patients who did not receive the 70-gene signature in different guideline delineated subgroups: group A (BR I, > 2 cm, N0) (*n* = 218); group B (BR II, > 1 cm, N0) (*n* = 3571); group C (BR I/II, any size, Nmi) (*n* = 1040); and group D (BR I/II, any size, N1a) (*n* = 1888). *P* values were calculated using a *χ*^2^ test for differences in categorical data

The 2399 patients (35%) who received a 70-GS, were slightly younger, had smaller tumors, and tumors of intermediate grade and less often positive lymph nodes compared with their counterparts in whom no 70-GS was used (Table 1). The use of the 70-GS increased from 24% of the eligible patients in 2013 to 51% of patients eligible for GEP use in 2016. This rising trend in 70-GS use was observed within all subgroups (A-D), but the clinically most significant increase in 70-GS use was seen in subgroup D (N1a patients): from 6% in 2013 to 50% of patients in 2016 (Fig. 2). The majority of patients who received the 70-GS were assigned to the 70-GS low-risk category: 68% in the whole group of patients and 85, 65, 73, and 73% in subgroups A, B, C, and D, respectively. The test result was adhered to in 91% of the overall study population: only in subgroup D compliance to the test was lower (85%; Supplementary Table 1).

**Table 1:** Patient and tumor characteristics according to 70-gene signature use in 6780 patients within the indicated area for 70-GS use (all younger than 70 years of age with ER+/HER2− invasive ductal carcinoma)

	70-GS not used *n* = 4381 *n* (%)	70-GS used *n* = 2399 *n* (%)	*P* value*
*Patient characteristics*			
Age at diagnosis (year), mean (SD)	57	56	<0.001**
Age categories (year)			
< 50	875 (20)	558 (23)	<0.001
50–59	1453 (33)	975 (41)	
60–69	2053 (47)	866 (36)	
Incidence year			
2013	1365 (31)	434 (18)	< 0.001
2014	1151 (26)	551 (23)	
2015	1032 (24)	545 (23)	
2016	833 (19)	869 (36)	
*Tumor characteristics*			
Pathological axillary status (pN)			
pN0 (i−, i+)	2076 (47)	1776 (74)	< 0.001
pNmi	739 (17)	301 (13)	
pN1a	1566 (36)	322 (13)	
*Pathological tumor size (cm), mean (SD)*	18	17	< 0.001**
Tumor size categories (cm)			
≤ 2	2980 (68)	1820 (76)	< 0.001
> 2	1387 (32)	573 (24)	
*NA*	14 (0.3)	6 (0.2)	
Invasive tumor grade			
Grade I	987 (23)	333 (14)	< 0.001
Grade II	3394 (77)	2066 (86)	

*70*-*GS* 70 gene-signature; *CT* chemotherapy; *BR* Bloom–Richardson grade; *N0* no axillary lymph node involvement; *Nmi* micrometastasis; *N1a* 1–3 ipsilateral positive axillary lymph nodes (at least one > 2 mm)

*χ^2^ test was used to compare frequencies in clinicopathological characteristics between patient who received the 70-GS (*n* = 2399) versus patients who did not receive the test (*n* = 4381)

**t test to assess the difference in mean age an tumor size (continuous variable)

### Chemotherapy Administration and 70-GS Use Within Guideline-Delineated Subgroups

For the whole study population, the proportion of patients who received adjuvant chemotherapy was lower when the 70-GS was used (30% vs. 46% of patients when the 70-GS was not used; *P* < 0.001). In a multivariable logistic regression analyses, 70-GS use remained associated with a decreased probability of administering adjuvant chemotherapy (odds ratio [OR] 0.65; 95% CI 0.57–0.73). For the whole study period, use of the 70-GS was associated with a nonsignificant decreased probability of administering chemotherapy in group A (OR 0.53; 95% CI 0.24–1.14, data not shown). In group B, the 70-GS was associated with a nonsignificant increased probability of receiving chemotherapy (OR 1.03; 95% CI 0.88–1.21, data not shown). For group C (Nmi) and group D (N1a), use of the 70-GS resulted in a significant decreased probability to receive chemotherapy (group C: 0.37, 95% CI 0.27–0.51, and group D: OR 0.21, 95% CI 0.15–0.29).

Different time trends for the interplay between 70-GS use and chemotherapy administration were observed for the four subgroups (Fig. 2a–d). In groups A and B, chemotherapy administration in the selection of patients in whom the 70-GS was deployed fluctuated during the study period within a limited range. Without using the 70-GS, the use of chemotherapy decreased over time and was rarely administered to N0 patients in 2016. Hence, while the use of the 70-GS in N0 patients (group A and B together) was associated with the administration of less chemotherapy in 2013 and 2014, more chemotherapy was administered in the recent years. In group C (Nmi), 70-GS use was associated with less chemotherapy administration in 2013 and 2014, while in more recent years this association was no longer observed. In N1a patients, the lower proportion of patients receiving chemotherapy when the 70-GS was deployed was consistent throughout the study period.

### Adjuvant Chemotherapy Administration and 70-GS Use by Age Categories

Subgroup analyses in patients delineated by age demonstrated a significant decrease in the administration of adjuvant chemotherapy in patients < 50 years and 50–59 years of age who received the 70-GS versus patients who did not receive the 70-GS (Supplementary Fig. 1a–c). Without the use of the 70-GS the likelihood of administering chemotherapy decreased with age: 79, 55, and 26% of patients were treated with adjuvant chemotherapy in the < 50, 50–59, and 60–69 years group, respectively. In multivariable logistic regression analysis, 70-GS use in patients < 50 years and in the 50–59 years group was independently associated with a decreased chance of chemotherapy administration (OR 0.17, 95% CI 0.13–0.23 and OR 0.53, 95% CI 0.43–0.65). In the older age group (60–69 years), a reverse association was observed; the 70-GS was independently associated with an increased chance of chemotherapy administration (OR 1.76, 95% CI 1.41–2.19). Age was not associated with a higher proportion of patients being assigned to a risk category based on the 70-GS test result: 68, 70, and 65% of patients were classified as low risk in the < 50, 50–59, and 60–69 years age groups, respectively.

## Discussion

In the present population-based study, in early-stage breast cancer patients who are considered candidates for GEP use, an increased use of the 70-GS was observed over time as well as a decrease in the administration of chemotherapy. For patients with lymph node positive disease and in younger patients, 70-GS use was associated with a consistent lower proportion of patients receiving adjuvant chemotherapy. In lymph node-negative patients, we observed a decrease in the use of chemotherapy over time, irrespective of 70-GS use.

The increased use of the 70-GS and the decrease in chemotherapy administration (from 49% of patients in 2013 to 23% in 2016) both demonstrate the growing restraint of Dutch clinicians to administer chemotherapy in the selection of patients identified as having luminal A-type breast cancers. The decline in chemotherapy administration coincides with recent international guideline recommendations.^3^ In 2015, the St. Gallen international consensus meeting stated that for patients with ER+/Her2− disease, a spectrum exists in degree of risk and responsiveness to chemotherapy and noted the increasing evidence for the use of multiparameter molecular test (e.g., the 70-GS and the 21-RS) to discriminate between “Luminal A-like” and “Luminal B-like” disease in order to better guide chemotherapy decisions. Interestingly, the results of our study indicate that this decline in chemotherapy use is not only explained by the use of GEPs, since also in patients in whom no 70-GS was deployed (i.e., in whom no difference between Luminal A or B disease was made) less chemotherapy was administered over time. Clinicians move away from administering chemotherapy in HR+/Her2−/N0 disease and apparently do not consider a multiparameter molecular test necessary to do so. These results are in line with a study conducted in the United States that examined trends in OncotypeDx deployment and chemotherapy use over the years 2013–2015. In the latter study, chemotherapy use in node-negative and micro-metastatic patients declined from 26.6 to 14.1%, and the reported decrease was independent of OncotypeDx use.^17^

In an earlier population-based study conducted in The Netherlands between 2011 and 2013, a period in which chemotherapy was commonly administered in patients with lymph-node negative disease, the 70-GS was independently associated with a decreased likelihood to receive chemotherapy.^14^ In the current study, which was conducted in more recent years, no independent association between 70-GS use and chemotherapy administration was observed in N0 patients as the administration of chemotherapy mostly decreased without 70-GS deployment. This more reluctant attitude among clinicians in administering chemotherapy in this patient category is supported by recent international chemotherapy recommendations as well as by recent studies that support omission of chemotherapy in clinical low-risk luminal type breast cancer patients.^3^^,^^8^,^10^ According to the MINDACT trial, there was no difference in 5-year DMFS in clinical low-risk patients assigned to the 70-GS high-risk category who did or did not receive chemotherapy, illustrating that there is no role for the 70-GS in clinical low-risk patients. This is different for lymph-node positive patients. In this category, a strong association between 70-GS use and less chemotherapy administration was observed and a lower proportion of patients in this category received a 70-GS in the current study. Because international guidelines are more cautious concerning 70-GS use in lymph-node positive patients, this is not surprising. However, in those lymph-node positive patients who did receive a 70-GS, less chemotherapy was administered. This finding also was reported by others and indicates a potential important benefit of 70-GS use in lymph-node positive patients, supported by the results of the MINDACT trial in which omission of chemotherapy in lymph-node positive patients (pN1a) with a 70-GS low-risk result appeared to be safe.^8^^,^^18^–^21^

Another important finding of our study was the age dependent effect of the 70-GS use. The reduction in the proportion of patients who received chemotherapy in association with 70-GS use was observed in the younger age categories (< 50 years and 50–59 years). Younger women more often present with more aggressive types of breast cancer compared with the older age category, and it is becoming clearer that tumor biology largely explains the impact of young age on breast cancer outcomes.^22^–^24^ In the present study, however, the aggressive molecular subtypes were not included, and the proportion of eligible patients who underwent genomic profiling and were assigned to the genomic high risk category was similar for all age groups. Notwithstanding the similar intrinsic molecular composition of the tumors in the age groups, chemotherapy was still substantially more often administered to young patients when the 70-GS was not used. A reversed relationship was seen in the older age category (60–69 years) as the use 70-GS was associated with an increased risk of receiving chemotherapy in the context of a limited tendency to administer chemotherapy without the 70-GS. The use of GEPs among young women with breast cancer apparently helps to reduce the tendency to “overtreat” young women.

A strength of this study is the nationwide character and the large cohort of breast cancer patients in whom the association of a GEP on the administration of adjuvant chemotherapy could be assessed. The retrospective design of this study is an important limitation of the study and prevents us from formulating statements that imply causality. During the study period, the national guideline of 2012 was effective suggesting the use of a GEP in ER+/HER2− breast cancer when there is doubt about the adjuvant chemotherapy benefit. Then again, international guidelines were formulated in the meantime providing different recommendations regarding the indications for gene expression profiling.^25^^,^^26^

## Conclusions

At a nationwide level in ER+/Her2− breast cancer patients, this study demonstrates a strong decrease in the administration of adjuvant chemotherapy over time without an adjustment in the national breast cancer guideline but in line with contemporary international consensus statements. For lymph node-negative patients, this decline in chemotherapy administration was independent of the 70-GS use, whereas in lymph node-positive disease and in younger patients, the 70-GS was associated with a significantly decreased likelihood that patients received adjuvant chemotherapy.

## Electronic supplementary material

Below is the link to the electronic supplementary material.

Supplementary material 1 (DOCX 12 kb)

Supplementary Fig. 1A-C Adjuvant chemotherapy use in patients who received the 70-gene signature versus patients who did not receive the 70-gene signature in relation to different age categories: <50 years (n = 1,433) (A), 50-59 years (n = 2,428) (B), and 60-69 years of age (n = 2,919) (C). *P* values were calculated using a *χ*^2^ test for differences in categorical data (EPS 651 kb)

## Data Availability

The data are available on request.
