# Supplementary material for: The Changing Role of Gene-Expression Profiling in the Era of De-escalating Adjuvant Chemotherapy in Early-Stage Breast Cancer
Source: Ann Surg Oncol. 2019 Jun 17;26(11):3495–501. doi: 10.1245/s10434-019-07511-8 (PMC6739278; doi:10.1245/s10434-019-07511-8)
Supplement: Supplementary file 1 — Supplementary material 1 (DOCX 12 kb) [file 10434_2019_7511_MOESM1_ESM.docx]

**Supplementary Table 1** The 70-GS test results and adherence rates for all patients in whom the 70-GS was used (n=2 399) and per subgroup A-D

|  | *N* | 70-GS low-risk (%) | 70-GS high-risk (%) | Unknown test result (%) | Adherence rate (%) |
| --- | --- | --- | --- | --- | --- |
| All patients  (A, B, C, D) | 2 399 | 68 | 31 | 1 | 91 |
| Group A  N0, BR I, >2cm | 109 | 85 | 15 | - | 94 |
| Group B  N0, BR II, >1cm | 1 667 | 65 | 34 | 1 | 91 |
| Group C  pNmi, BR I/II | 301 | 73 | 26 | 1 | 90 |
| Group D  pN1a, BR I/II | 322 | 73 | 25 | 2 | 85 |
| 70-GS; 70 gene signature, CT; chemotherapy, BR; Bloom-Richardson grade, N0; no axillary lymph node involvement, Nmi; micro-metastasis, N1a: 1-3 ipsilateral positive axillary lymph nodes (at least one >2mm)  Adherence rates were calculated by dividing the sum of patients with a low-risk test result in whom adjuvant CT was omitted and patients with a high-risk test result who received adjuvant CT by all patients with a known test result. | | | | | |
